# Who Should Be Targeted for the Prevention of Birth Defects? A Latent Class Analysis Based on a Large, Population-Based, Cross-Sectional Study in Shaanxi Province, Western China

**DOI:** 10.1371/journal.pone.0155587

**Published:** 2016-05-16

**Authors:** Zhonghai Zhu, Yue Cheng, Wenfang Yang, Danyang Li, Xue Yang, Danli Liu, Min Zhang, Hong Yan, Lingxia Zeng

**Affiliations:** 1 Department of Epidemiology and Biostatistics, School of Public Health, Xi’an Jiaotong University Health Science Center, Xi’an, Shaanxi, P.R. China; 2 Department of Nutrition and Food Safety Research, School of Public Health, Xi’an Jiaotong University Health Science Center, Xi’an, Shaanxi, P.R. China; 3 Department of Maternal and Child Health Center, the First Affiliated Hospital, Xi’an Jiaotong University Health Science Center, Xi’an, Shaanxi, P.R. China; Tabriz University of Medical Sciences, ISLAMIC REPUBLIC OF IRAN

## Abstract

**Background:**

The wide range and complex combinations of factors that cause birth defects impede the development of primary prevention strategies targeted at high-risk subpopulations.

**Methods:**

Latent class analysis (LCA) was conducted to identify mutually exclusive profiles of factors associated with birth defects among women between 15 and 49 years of age using data from a large, population-based, cross-sectional study conducted in Shaanxi Province, western China, between August and October, 2013. The odds ratios (ORs) and 95% confidence intervals (CIs) of associated factors and the latent profiles of indicators of birth defects and congenital heart defects were computed using a logistic regression model.

**Results:**

Five discrete subpopulations of participants were identified as follows: No folic acid supplementation in the periconceptional period (reference class, 21.37%); low maternal education level + unhealthy lifestyle (class 2, 39.75%); low maternal education level + unhealthy lifestyle + disease (class 3, 23.71%); unhealthy maternal lifestyle + advanced age (class 4, 4.71%); and multi-risk factor exposure (class 5, 10.45%). Compared with the reference subgroup, the other subgroups consistently had a significantly increased risk of birth defects (ORs and 95% CIs: class 2, 1.75 and 1.21–2.54; class 3, 3.13 and 2.17–4.52; class 4, 5.02 and 3.20–7.88; and class 5, 12.25 and 8.61–17.42, respectively). For congenital heart defects, the ORs and 95% CIs were all higher, and the magnitude of OR differences ranged from 1.59 to 16.15.

**Conclusions:**

A comprehensive intervention strategy targeting maternal exposure to multiple risk factors is expected to show the strongest results in preventing birth defects.

## Introduction

Birth defects affect 3% of all babies, cause 2.7 million neonatal deaths, and result in approximately 3.2 million birth defect-related disabilities every year. They have a significant effect on individuals, families, health-care systems and societies [1]. Birth defects are particularly serious in low- and middle-income countries due to consanguineous marriage, advanced maternal age, migration and poverty [2]. The prevalence of birth defects has been increasing annually in China, which is the largest developing country [3–7], and this has resulted in substantial economic burdens for both the family of the person with birth defects and society. In China, the average lifetime economic burdens of a new case of Down syndrome from the family and societal perspectives are US$47,000 and US$55,000, respectively [8]. In 2008, 11% of all deaths among children (< 5 years) were from birth defects, and this proportion has been predicted to continue increasing in the long term [9]. Therefore, studying the causes and determinants of birth defects to develop an effective prevention program based on the Chinese population is a public health priority. Such a program may be one important way to promote the target of achieving a two-thirds reduction in child mortality levels by 2015, which was proposed in 1990 as part of the Millennium Development Goals [10].

Since 2010, WHO has been encouraging countries to develop primary prevention and health promotion strategies for birth defects and to raise awareness of their effects [11, 12]. However, there are many challenges in the process because of the wide range of types and causes of birth defects, whose pathogenesis is unclear. Birth defects are a diverse group of structural or functional disorders, including metabolic disorders, which can be caused by genetic factors; maternal factors, including maternal infections such as syphilis and rubella, nutritional status and socioeconomic factors; teratogens (i.e., harmful environmental factors); or interactions among these risk factors [1]. Even now, the causes of approximately 50% of birth defects cannot be ascertained [13]. Therefore, identifying modifiable causes and risk factors is a hot topic in the study of birth defects prevention.

Previous studies have suggested that maternal factors from multiple domains are associated with birth defects. While advanced maternal age [14]; maternal stress [15, 16]; low maternal education level [17]; adverse life style, such as maternal smoking [18, 19]; and exposure to harmful environmental factors [20–22] are associated with an increased risk of birth defects, folic acid supplementation appears to be protective [23]. Although these associations have been studied extensively by incorporating factors into a multiple regression model, very limited evidence is available to fully explain the complex linkages of birth defects and the patterns of associated factors and to identify the high-risk subpopulations. Many of these associated factors tend to occur simultaneously and are likely inter-related within some individuals, and they cannot be examined due to some methodological challenges (i.e., statistical power issues), which leads to the conclusion that specific combinations of risk factors are no more predictive of outcomes than a total number of factors would be [24].

The co-occurrence patterns of associated factors must be examined because this information could aid in the development of intervention strategies that are relevant to the prevention of birth defects among women of childbearing age. In designing health promotion strategies, it is important to understand the many factors that women are simultaneously experiencing and that are unlikely to occur in an entirely uniform manner in the population. For example, if most women with poor dietary intake are also smoking and experiencing stress, narrow intervention strategies specifically targeting nutrition-related behavior changes (e.g., periconceptional folic acid, salt iodization or iodine supplements) may not be effective in helping these women. However, using the same intervention strategy related to all modifiable risk factors in the entire population is neither efficient nor cost effective, particularly in China, which has limited per capita financial and temporal resources [25]. Indeed, the “infinite combinations of risk factors with which patients may present” is a common challenge that public health professionals have to face when developing programs for multiple risk factor interventions [26]. These examples highlight the need for a basic understanding of the patterns of overall factors associated with birth defects in women of childbearing age.

Latent class analysis (LCA) is conceptually similar to cluster analysis, which is based on a measurement model much like factor analysis, and is one useful approach to address these needs in the field. LCA is a data-driven approach positing that there are underlying homogeneous, mutually exclusive subgroups or latent classes existing within a heterogeneous population, but a true subgroup membership of individuals who share similar characteristics is unknown and can only be inferred through a measurement model of a set of observed categorical indicators. Therefore, instead of subgrouping every possible factor combination, LCA helps to address the complexity of birth defects-related factor patterning and to capture the most parsimonious, meaningful key patterns of factors, while accounting for measurement error [27].

LCA is increasingly being applied to identify memberships of study populations based on modeling multidimensional factors, including weight-related health behaviors [28], socioeconomic position [29], academic and behavior problems [30], executive function development [31], and dietary patterns of pregnant women [32, 33], which may be used to study factor profiles associated with birth defects. To address this gap in the literature and to offer the right program to the right individuals or target limited resources to the highest-risk subgroups, we have used this latent membership perspective in the present study to identify distinct classes of birth defect-related factors among Chinese women of childbearing age (15 to 49 years), in a large population-based sample of individuals living in western China; to describe the prevalence and constructs of classes; and to examine differences in how factor profiles and single factors are related to birth defects.

## Materials and Methods

### Study design and subjects

A population-based, cross-sectional survey was conducted between August and October, 2013. The subjects were Chinese women of childbearing age (15 to 49 years) living in Shaanxi, a major province in western China with a population of 40 million that is undergoing rapid economic change. The inclusion criteria for the women were a pregnancy that ended between August 2011 and August 2013 and a birth that occurred at a gestational age of ≥28 weeks. Women were excluded if they were pregnant during the time of the survey or had a serious illness, such as cancer or cardiovascular disease. A stratified, multistage, random sampling method was used to obtain the sample. Women were recruited from 10 urban districts and 20 rural counties that were randomly selected according to the proportion of urban to rural residents and the fertility level in Shaanxi Province. In urban areas, 3 subdistricts were randomly selected from each sampled district, 6 communities were randomly selected from each sampled subdistrict and the urban women were selected from each sampled community. In rural areas, for each sampled county, 6 townships were randomly selected, and 6 villages were randomly selected from the sampled townships. Finally, the rural women were randomly selected from the sampled villages. To gain a larger sample size and detect more birth defects in the population, we assumed that the prevalence of birth defects in Shaanxi Province was 1% when estimating sample size, while the reported prevalence of birth defects is 1.4% according to the results of a population-based, cross-sectional survey conducted in 128 townships in 16 counties/districts in Shaanxi Province between 2008 and 2009. A sample size of 30,000 was estimated assuming a prevalence of birth defects of 1%, a relative error of 20%, a 2.0 designing deficiency, two-tailed α = 0.05, and an expected response rate of 90%. The proportion of urban to rural residents in the sample was determined to be approximately 50%, which was close to the proportion of urban to rural population in Shaanxi Province.

### Data collection/ associated factors

All women were interviewed in person by trained interviewers who used a standard questionnaire comprising four sections. The first part was designed to collect information on maternal sociodemographic characteristics, including age, ethnicity, education, marital status, address of residence, and occupation at the time of the survey. The second part was primarily about the outcomes of each woman’s most recent pregnancy, which was identified as the woman’s most recent live birth or stillbirth at a gestational age of ≥28 weeks, to confirm birth defects. Birth defects were classified according to the International Classification of Diseases Clinical Modification Codes, tenth edition, (ICD-10) as congenital malformations, deformations, and chromosomal abnormalities(Codes Q00–Q99). First, we asked participants who reported birth defects to provide their medical records. Second, for participants who either did not report birth defects or reported them but did not provide their medical records, we used one of two measures to identify cases. Either we checked the surveillance system of birth defects at the corresponding hospital where that participant had delivered her baby or the interviewers performed physical examinations of the infants to detect apparent structural defects and took pictures that would be reviewed and diagnosed by experts. Third, we obtained data on the women’s reproductive history by asking about their previous pregnancy outcomes, including induced abortions, miscarriages, stillbirths, preterm deliveries and any birth defects associated with previous pregnancies. Fourth, we focused on maternal exposure to periconceptional risk factors associated with birth defects during the most recent pregnancy, which covered the following topics: lifestyle, illnesses, periconceptional stress, exposure to harmful factors in the periconceptional environment, family history of diseases and periconceptional folic acid supplementation. Periconceptional was defined as the period from 3 months prior to pregnancy through pregnancy. Information on lifestyle included alcohol use (drinking Chinese wine or beer at a frequency of more than once per week); smoking (smoking cigarettes continuously or cumulatively for three months or more); passive smoking (defined as family members who smoked cigarettes while living together with the women); and drinking strong tea and/or coffee (at a frequency of more than once per day). Maternal diseases were defined as colds; fevers; and pregnancy complications, including pregnancy-induced hypertension and diabetes mellitus. Information about maternal stress during the periconceptional period was obtained by asking three questions: “Have you experienced serious life crisis events, such as marital discord, bereavement, unemployment or major diseases?”, “Have you experienced domestic violence?” or “Have you experienced low spirits, irritability, or any suicidal thoughts?”. As long as there was one “yes” answer, the woman would be defined as having experienced stress during the periconceptional period. Harmful environmental factors were defined as either the women’s residence during the most recent pregnancy being less than 3 kilometers, with the content of pollutants varying significantly as the distance changed, from industrial production sites that may discharge pollutants, such as coal mines, paper mills, cement plants, and coal-fired power plants, or exposure to any radioactive examination or chemical pesticide, including insecticides, herbicides and rodenticides, during the periconceptional period. A family history of diseases that might be related to birth defects was also evaluated, including incidences of birth defects and Keshan disease (an endemic cardiomyopathy named after Keshan county in Heilongjiang Province, northeast China, where the symptoms were first noted). Women were asked to report their use of periconceptional nutritional supplements, including folic acid and other micronutrients.

### Statistical analysis

The analyses reported here were confined to women with single births because multiple births increase the risk of birth defects [34, 35]. Only isolated congenital malformations (ICD-10) were included when calculating the prevalence of birth defects. When one child was diagnosed with multiple defects, it was recorded as one case. The prevalence of birth defects was calculated as the number of birth defect cases divided by the total number of stillbirths and live births.

All factors associated with birth defects were coded as dichotomous indicators (e.g., no = factor not present, yes = factor present). Although many factors in this study were dichotomous by nature, several continuous measures of factors were converted into dichotomous factors by logical or previously established cut-offs. Maternal education level and age at the time of the interview were dichotomized by cut-offs 9 and 30 years, respectively, which were selected based on the conditions of most reproductive-aged women having a low education level and being of a young age reported in previous studies [17, 36]. We assessed the presence or absence of factors associated with birth defects during the periconceptional period in multiple domains related to demographic characteristics, lifestyle, family history of diseases, maternal diseases and stressors, exposure to harmful environmental factors, previous adverse pregnancy outcomes, and maternal periconceptional folic acid supplementation. Within certain domains, we chose to combine two or more factors to assess whether any domain indicator was present in the broader conceptual domain, which is a strategy used in previous studies [30, 37, 38]. For example, “smoking or passive smoking”, “drinking alcohol” and “drinking strong tea or coffee” were combined to create a single risk indicator within the unhealthy lifestyle domain (see Table 1).

**Table 1:** Summary of factors associated with birth defects in Shaanxi Province, 2011–2013 (N = 29,547).

Indicator	Rules for creation of risk factor	Prevalence (%)
No folic acid supplementation in the periconceptional period	No folic acid supplementation from 3 months before to 3 months after conception*	87.65
Unhealthy lifestyle	Drinking alcohol, drinking strong tea and coffee, or smoking and passive smoking	60.54
Previous adverse pregnancy outcomes	A History of abnormal pregnancy, miscarriage, or birth defects	19.45
Family history of diseases	A History of Keshan disease, or birth defects in first or second-degree relatives	6.25
Exposure to harmful environmental factors	Living near factories that pollute heavily or exposure to any pesticide or radioactive environment	32.19
Maternal stress	Exposure to stressors, including domestic misfortune, low spirits and marital discord	4.32
Low maternal education level	Maternal education level of ≤9 years	62.16
Advanced maternal age	Maternal age >29 years	30.38
Maternal diseases	Experiencing diseases such as colds, fevers, or pregnancy complications during the period from 3 month preconception through pregnancy	36.83

*The provincial government has provided folic acid free of charge for reproductive-aged women from 3 months before to 3 months after conception.

We used LCA to define groups of women with similar factor profiles across the 9 indicators associated with birth defects. Latent class models with 2 or more classes were fitted to items measuring different aspects of the women’s factor profiles. The estimation was performed using the maximum likelihood with expectation-maximization algorithm. Criteria used to select the final LCA model included the G^2^ (G-squared test statistic), the Bayesian information criterion (BIC) and the Akaike information criterion (AIC), with lower values indicating a better model fit [39]. However, the BIC performs slightly better than the AIC when the sample size is larger than 1000 [40]. In this study, we relied on these information criteria as well as on the interpretability of the findings to determine the optimal number of factor-latent profiles. After selecting the final LCA model, the posterior probability of belonging to each factor profile could be obtained for each woman.

After the most parsimonious latent class model was determined, the odds ratios (ORs) with their 95% confidence intervals (CIs) were used to estimate the strength of the associations between factor profiles and either birth defects or congenital heart defects using the classify-analyze approach. To compare the results of exploring the causes of diseases using traditional multivariable analysis, the ORs and 95% CIs of all indicators of birth defects and congenital heart defects were computed using a logistic regression model, in which all indicators described in Table 1 were put into the model simultaneously.

All LCA models were fitted using Mplus (V.5.2; Muthen & Muthen, Los Angeles, California, USA), while SPSS 20.0 software (Chicago Illinois, USA) was used for the logistic regressions. A P value of <0.05 was considered statistically significant using a 2-tailed test.

### Ethics Statement

The study was conducted in accordance with the Declaration of Helsinki and was approved by the Human Research Ethics Committee of the Xi'an Jiaotong University Health Science Center (No. 2012008). Written informed consent was obtained from all participants after the nature of the study had been explained. Participants who were 15 to 17 years old during the study period and had therefore not reached the minimum legal marriageable age of 20 for females and 22 for males in China also provided oral informed consent for themselves, and their mothers-in-law provided written consent on behalf of these participants. There was no need to obtain informed consent from the next of kin, caretakers, or guardians on behalf of the minors/children enrolled in our study because all information about the infants was obtained from their mothers.

## Results

### Demographic characteristics

In total, 30,027 women were interviewed, and 29,608 (98.60%) singleton births were included in this study. Among them, 61 women were excluded for incomplete or missing data regarding the factors associated with birth defects, and 29,547 observations were used for the final analysis. The demographic characteristics are shown in Table 2. Of the women analyzed, the overall mean [standard deviation (SD)] age was 28.33 (4.81) years (range, 16 to49 years); 99.27% (29,333) were ethnically Han Chinese; 62.16% (18,635) had ≤9 years of education; 97.65% (28,852) were married; and 79.66% (23,538) were from rural areas. Regarding maternal occupation, 64.11% (18,942) of the women had occupations related to farming.

**Table 2:** Sociodemographic characteristics of the participants from Shaanxi Province, 2011–2013 (N = 29,547).

Characteristics	Frequency (%)
Age in years, mean (SD)	28.33 (4.81)
Han ethnicity	29,333 (99.27)
Education	
≤6 years	3,670 (12.42)
7–9 years	14,695 (49.73)
>9 years	11,182 (37.84)
Married	28,852 (97.65)
Residence	
Rural	23,538 (79.66)
Urban	6,009 (20.34)
Occupation	
Farmer	18,942 (64.11)
Migrant worker	2,030 (6.87)
Business	3,321 (11.24)
Government staff	3,929 (13.30)
Scientific or technical personnel	1,325 (4.48)

### Prevalence of birth defects and distribution of factors associated with birth defects

The prevalence of birth defects was 1.80% (532/29,547), and congenital heart defects were the most common type of birth defect, with a prevalence of 0.58% (172/29,547). As shown in Table 1, the prevalences of factors associated with birth defects, in decreasing order, were: “No folic acid supplementation in the periconceptional period” (87.65%); “Low maternal education level” (62.16%); “Unhealthy lifestyle” (60.54%); “Maternal diseases” (36.83%); “Exposure to harmful environmental factors” (32.19%); “Maternal advanced age” (30.38%); “Previous adverse pregnancy outcomes” (19.45%); “Family history of diseases” (6.25%); and “Maternal stress” (4.32%).

### Multivariable logistic regression analysis of factors associated with birth defects and congenital heart defects

As shown in Table 3, all factors in the model except for unhealthy lifestyle and advanced age were significantly associated with an increased risk of birth defects. “Previous adverse pregnancy outcomes” showed the strongest association with birth defects. The OR and 95% CI were 15.94 and 12.86–19.75, respectively, while the ORs for the other factors ranged from 1.00 to 1.50. Only “No folic acid supplementation in the periconceptional period”, “Previous adverse pregnancy outcomes” and “Maternal diseases” were associated with an elevated risk of congenital heart defects.

**Table 3:** Multivariable logistic analyses of factors associated with overall birth defects and congenital heart defects in Shaanxi Province, 2011–2013.

Factors	Overall birth defects		Congenital heart defects	
	OR(95%CI)	P	OR(95%CI)	P
No folic acid supplementation in the periconceptional period	1.37(1.00,1.88)	0.047	1.96(1.05,3.64)	0.034
Unhealthy lifestyle	1.06(0.88,1.28)	0.531	1.24(0.88,1.74)	0.212
Previous adverse pregnancy outcomes	15.94(12.86,19.75)	<0.001	19.28(12.79,29.05)	<0.001
Family history of diseases	1.36(1.03,1.81)	0.033	1.18(0.72,1.95)	0.506
Exposure to harmful environmental factors	1.21(1.01,1.45)	0.037	1.27(0.93,1.73)	0.128
Maternal stress	1.50(1.10,2.04)	0.010	1.45(0.88,2.41)	0.146
Low maternal education level	1.23(1.02,1.49)	0.035	1.00(0.72,1.39)	0.983
Advanced maternal age	0.97(0.81,1.17)	0.758	1.23(0.90,1.68)	0.191
Maternal diseases	1.32(1.11,1.58)	0.002	1.93(1.41,2.63)	<0.001

Abbreviations: CI, confidence interval; OR, odds ratio.

### Prevalence and characterization of five latent classes

Table 4 shows consistently decreasing G^2^ and AIC values of the 2 through 6 class models; however, based on the interpretability and the lowest BIC (BICs: 4 classes = 264,412, 5 classes = 264,394, 6 classes = 264,450), we determined that the 5-class model best described the different indicators associated with birth defects in this study.

**Table 4:** Fit statistics for LCA models with 2 to 6 classes.

No. of classes	G^2^	df	p	AIC	BIC
2	2023	492	<0.001	265352	265509
3	1382	482	<0.001	264731	264971
4	720	472	<0.001	264088	264412
**5**	**598**	**462**	**<0.001**	**263987**	**264394**
6	551	452	<0.001	263961	264450

For the 5-class solution, conditional item-response probabilities are shown in Table 5. Each column represents the probability of individuals in a particular latent class reporting each indicator. Higher probabilities (>0.50) signify defining characteristics of factor profile membership. As shown in Table 5, 21.37% of the women were expected to belong to latent class 1, which was characterized by a low probability of reporting each indicator except for “No folic acid supplementation in the periconceptional period”, which had a high probability in all latent classes. These women would be referred to as the “reference” latent class. Women in latent class 2 (39.75%) were characterized by a high probability of reporting an education level ≤9 years and an unhealthy lifestyle. Women in latent class 3 (23.71%) were characterized by a high probability of reporting an education level of ≤9 years, an unhealthy lifestyle and diseases. Using the same principle, class 4 comprised the smallest latent class (4.71%) and was characterized by a high probability of reporting an unhealthy lifestyle and advanced maternal age. Finally, latent class 5 (10.45%) comprised women who reported having experienced most risk indicators, including an unhealthy lifestyle, maternal exposure to harmful environmental factors, low maternal education level, maternal age older than 29 years and maternal diseases.

**Table 5:** Five-latent-status model of factors associated with overall birth defects and congenital heart defects in Shaanxi Province, 2011–2013 (N = 29,547).

	Latent classes				
Item-response probabilities	Class 1 reference	Class 2^a^	Class 3^b^	Class 4^c^	Class 5^d^
No folic acid supplementation in the periconceptional period	0.79*	**0.93**	**0.91**	**0.66**	**0.96**
Unhealthy lifestyle	0.47	**0.55**	**0.71**	**0.55**	**0.76**
Previous adverse pregnancy outcomes	0.08	0.12	0.16	0.36	0.45
Family history of diseases	0.02	0.04	0.04	0.14	0.14
Exposure to harmful environmental factors	0.22	0.22	0.41	0.33	**0.51**
Maternal stress	0.00	0.00	0.09	0.04	0.11
Low maternal education level	0.15	**1.00**	**0.55**	0.29	**0.83**
Advanced maternal age	0.23	0.38	0.00	**0.56**	**0.53**
Maternal diseases	0.21	0.21	**0.59**	0.40	**0.52**
Latent status prevalence	0.20	0.32	0.24	0.09	0.16
Proportion of women	0.21	0.40	0.24	0.05	0.10
Overall birth defects					
OR(95%CI)	ref	1.75(1.21,2.54)	3.13(2.17,4.52)	5.02(3.20,7.88)	12.25(8.61,17.42)
P	-	0.003	<0.001	<0.001	<0.001
Congenital heart defects					
OR(95%CI)	ref	3.34(1.30,8.59)	8.89(3.54,22.32)	17.46(6.51,46.85)	28.40(11.44,70.50)
P	-	0.012	<0.001	<0.001	<0.001

Abbreviations: CI, confidence interval; OR, odds ratio.

*Item-response probabilities > 0.50 in bold to facilitate interpretation. Each latent class reported no folic acid supplementation in the periconceptional period with a high probability (>0.50). Women in latent class 1 (21.37%) were solely characterized by a high probability of reporting no folic acid supplementation in the periconceptional period.

^a^ Women in latent class 2 (39.75%) were characterized by a high probability of reporting an education level of ≤9 years and an unhealthy lifestyle.

^b^ Women in latent class 3 (23.71%) were characterized by a high probability of reporting an education level of ≤9 years, an unhealthy lifestyle and diseases.

^c^ Women in latent class 4 (4.71%) were characterized by a high probability of reporting an unhealthy lifestyle and maternal age older than 29 years.

^d^ Women in latent class 5 (10.45%) were characterized by a high probability of reporting most risk indicators, including an unhealthy lifestyle, maternal exposure to harmful environmental factors, low maternal education level, maternal age older than 29 years and diseases.

### Association between latent class membership and birth defects or congenital heart defects

The lower section of Table 5 shows the ORs and 95% CIs of the latent class memberships with birth defects and congenital heart defects. Compared with the “reference” (No folic acid supplementation in the periconceptional period) membership, class 2 (OR = 1.75, 95% CI: 1.21–2.54); class 3 (OR = 3.13, 95% CI: 2.17–4.52); class 4 (OR = 5.02, 95% CI: 3.20–7.88); and class 5 (OR = 12.25, 95% CI: 8.61–17.42) were consistently significantly associated with an increased risk of birth defects. Concerning congenital heart defects, the ORs and 95% CIs were all higher, and the magnitude of OR differences ranged from 1.59 to 16.15. In particular, the women in class 5, which was characterized by multiple risk indicators, exhibited approximately 28 times the risk of having a baby with a congenital heart defect when the “No folic acid supplementation in the periconceptional period” membership was used as the reference.

## Discussion

### Main findings

In a large population-based sample of reproductive-aged women from Shaanxi Province in western China, where the prevalence of birth defects is 1.80% (532/29,547), and the prevalence of the most common type (congenital heart defects) is 0.58% (172/29,547), five distinct latent subgroups (No folic acid supplementation in the periconceptional period as reference (21.37%); Low maternal education level + unhealthy lifestyle (39.75%); Low maternal education level + unhealthy lifestyle + disease (23.71%); Unhealthy maternal lifestyle + advanced age (4.71%); Multi-risk factor exposure (10.45%) that adequately accounted for variations in the co-occurrence patterns of 9 associated indicators in terms of birth defects were identified using LCA. Each subgroup exhibited a unique latent profile of associated indicators. Compared with the study of associated indicators in isolation, these profiles evaluated individuals more holistically and suggested the manner in which multiple factors were organized within individuals. Memberships in these subgroups were significantly associated with birth defects and congenital heart defects, which were measured separately in logistic regression models for further analysis, and the results showed that the association with the latent subgroup characterized by multiple factors was stronger than that with the other latent subgroups. Therefore, these findings provide new insights into the health needs of this population and refine our understanding of the intersection of birth defect-related factors so that we can develop successful intervention programs tailored for and targeted at high-risk population subgroups characterized by the corresponding factor profiles illustrated in this analysis.

### The prevalence of birth defects and associated factors

The prevalence of birth defects in our study sample was 1.80%, which was slightly higher than the prevalence of 1.43% reported in a population-based survey conducted between 2009 and 2010 [41]. An upward trend in the prevalence of birth defects over time has also been detected in other provinces in China [3–7]. The prevalence of congenital heart defects was found to be the highest (0.58%) in this study, which is consistent with previous studies in China [5, 6, 41, 42] but differs from studies in Shanxi [36, 43], Inner Mongolia [41, 44] and Chongqing [45].

Multivariable logistic regression analyses showed that low maternal education level, maternal diseases, no folic acid supplementation in the periconceptional period, exposure to harmful environmental factors and periconceptional stress were significantly associated with a higher risk of birth defects, which is consistent with the findings of other studies [15–17, 20–23, 36]. However, only three indicators including no folic acid supplementation in the periconceptional period, previous adverse pregnancy outcomes and maternal diseases were significantly associated with congenital heart defects. We did not find any association between maternal age older than 29 years or an unhealthy lifestyle and either birth defects or congenital heart defects. This discordant result may be due to the different subjects and measurements in terms of advanced age and unhealthy lifestyle [14, 18, 19]. Compared with the results from multivariable logistic analyses, those obtained from the classify-analyze approach identified advanced maternal age and an unhealthy lifestyle as significantly associated with birth defects and congenital heart defects, which may indicate that some factors could play a role in the associations through interactions with other related factors.

When we used “No folic acid supplementation in the periconceptional period” membership as the reference, we found that class 2 (characterized by a low maternal education level and an unhealthy lifestyle); class 3 (characterized by a low maternal education level, an unhealthy lifestyle and diseases); class 4 (characterized by an unhealthy lifestyle and advanced age); and class 5 (characterized by multiple risk indicators, including an unhealthy lifestyle, exposure to harmful environmental factors, a low education level, advanced age and diseases) were all consistently significantly associated with an increased risk of birth defects or congenital heart defects. Not surprisingly, the observation that this result differed from that of only three indicators significantly associated with congenital heart defects in multivariable logistic regression analyses demonstrated the utility of LCA in understanding the complex relationships among factors and outcomes, such as those observed in the current analyses.

It is interesting that the effects of different indicator profiles on congenital heart defects appear to be stronger than the effects on any birth defects, and the magnitude of OR differences ranged from 1.59 to 16.15. This might be because different types of birth defects are caused by different determinants. For example, advanced maternal age is more highly associated with numerical chromosomal aberrations, such as trisomy 21 (Down syndrome) [46], and folic acid supplementation during the periconceptional period, alone or in combination with vitamins and minerals, is more strongly associated with neural tube defects [47], while no specific factors to date have been identified for the occurrence of congenital heart defects. However, our results showed that membership in the class 5 subgroup (Multi-risk factor exposure) was associated with a 28-fold increased risk compared with the “reference” subgroup, indicating that congenital heart defects are caused by the complex interactions between demographic characteristics, lifestyle factors, harmful environmental exposures and genomic susceptibilities [48]. Regardless of the explanation, this finding highlights important issues concerning the potential need to distinguish different types of birth defects when evaluating the effectiveness or validity of prevention programs.

### Five discrete subpopulations and their significance for public health strategy makers

Although the findings from multivariable logistic regression analyses were helpful in understanding how multiple factors are associated with birth defects and highlighted that overall associated factors should be the focus of prevention efforts, they provided little information on how we should design future intervention strategies or whom we should target. These findings simply imply that the most appropriate, effective preventive approach for reducing the risk of birth defects would be to try to reduce the number of associated indicators for all women, regardless of how an individual’s entire spectrum of associated indicators interact to predict negative outcomes or which associated indicators are present in an individual’s life. By contrast, the five meaningful latent profiles derived by LCA provided a complete picture of the intersection of associated indicators and birth defects among these reproductive-aged women in terms of both the number of indicators present in each factor profile subgroup and which associated factors dominated the profile, meaning that they will fit well into a holistic/ecological framework for the development of intervention strategies.

Specifically, the item-response probability of folic acid supplementation in the periconceptional period was significantly smaller for all latent classes, indicating that “No folic acid supplementation in the periconceptional period” is very common among women of childbearing age in the Shaanxi Province. Despite the fact that WHO has recommended folic acid supplementation to prevent neural tube defects and that the provincial government has provided folic acid tablets free of charge since 2009, our results still suggest that more health declarations or intervention strategies for improving folic acid supplementation in the periconceptional period should be provided in Shaanxi Province. However, promoting such strategies with the aim of reducing the risk of birth defects may only be efficient and practical for members of the “reference” subgroup, which was predicted to represent 20.91% of the sample, because this subgroup was solely characterized by the indicator “No folic acid supplementation in the periconceptional period”; other latent classes were characterized by more than one indicator. The largest subgroup of women, comprising 39.75% of the sample, was class 2. This class was defined by a high probability of reporting an education level of ≤9 years and an unhealthy lifestyle, and a lower probability of reporting other factors; therefore, a more straightforward approach to providing periconceptional health education for promoting healthy lifestyles, such as avoiding passive smoking, may be appropriate for these women. Nearly a quarter of the sample comprised class 3 individuals, who were highly likely to experience diseases based on the class 2 factor profile. Members of this class may benefit most from the prevention or seasonable treatment of diseases that do not focus solely on improvements lifestyle [18, 49], or perhaps these individuals may require more antenatal care (beyond the WHO recommendations) to mitigate their risk for birth defects. A total of 4.71% of the sample belonged to class 4, which was characterized by advanced maternal age and an unhealthy lifestyle, suggesting that intervention programs designed for class 2 members could also be implemented to target this group effectively. Unlike the other classes, class 5, which comprised 10.45% of the sample, exhibited a high probability of engaging in multiple risk indicators, including an unhealthy lifestyle, exposure to harmful environmental factors, a low education level, advanced maternal age and diseases. As we hypothesized, we found that membership in this class was most strongly associated with birth defects and congenital heart defects compared with the “reference” class, suggesting that more risk indicators mean a higher risk for birth defects and demonstrating that a combination of risk indicators is greater than the sum of single indicators. Given the co-occurrence of associated factors across multiple domains, intensive combination interventions directed at these factors simultaneously may be needed for these high-risk memberships of significance prevalence.

### Limitations and strengths

Despite the interesting implications of these findings, several limitations of our study are important to address. First, because our participants were Chinese women of childbearing age(15 to 49 years), from Shaanxi Province in western China, these findings may be limited in terms of their generalization to other ethnic or racial populations. Second, the generalizability of our study may also be influenced by the indicators used in LCA because the number, prevalence and profiles of classes may differ if other associated indicators (e.g., diet) are considered in concert with the indicators that we focused on in the present study. Third, although dichotomizing associated factors are commonly used in LCA and may be helpful in interpreting and communicating the findings, this may result in some loss of information from categorizing the data in this way. However, most associated factors used in this study were categorical by nature except for maternal age and education level, while in some cases, receiver operating characteristic curves or latent profile analysis may aid in addressing such issues [50, 51]. Fourth, the associations between indicator profiles and any birth defects or congenital heart defects examined using the classify-analyze approach may be attenuated and biased to the extent that there is classification error, but the large sample size, OR values and practice in previous studies indicate that the effects of indicator profiles on birth defects or congenital heart defects are accepted [27, 52]. Fifth, the recall bias could not be eliminated completely due to the limitations of the cross-sectional design, although we used a series of effective measures to control biases as much as possible. Finally, this study only identified indicator profiles that were associated with negative outcomes at one point in time due to the cross-sectional study design; statistical techniques, such as latent transition analysis, can be used in future studies to capture changes in indicator profiles longitudinally [53].

This study also had several strengths. Compared with the data from hospital-based birth defect surveillance, the large, population-based sample used in this analysis provided more factors associated with birth defects, such as lifestyles, nutritional factors, environmental risk exposures, maternal illnesses, and genetic factors, to more accurately determine the prevalence of birth defects in a population and to inform the development and evaluation of prevention strategies [54, 55]. This study also demonstrated the utility of applying LCA to research on birth defect-related factors and identified five latent subgroups of reproductive-aged women marked by different profiles of indicators, suggesting possible high-risk targets for future interventions and confirming the importance of prevention efforts for reproductive-aged women based on comprehensive profiles of associated factors.

## Conclusions

Using LCA, we identified five latent subgroups characterized by the intersection of multiple recognized causes and determinants of birth defects among Chinese women of childbearing age (15 to 49 years) from Shaanxi Province in western China. More than 80 percent of women of childbearing age in Shaanxi Province do not receive folic acid supplementation during the periconceptional period and exposure to multiple risk factors associated with birth defects and congenital heart defects is high among these women, although the factors were co-occurring in different profiles. The combined effects of maternal exposure to multiple risk factors raises the risk of birth defects and congenital heart defects among offspring, and this risk is significantly higher than the simple summation of the risks from single factors. A comprehensive intervention strategy targeting maternal exposure to multiple risk factors will contribute to the primary prevention of birth defects, particularly for high-risk subgroups, which are expected to show the strongest response.
